# Evaluation of an obstetric and neonatal care upskilling program for community health workers in Papua New Guinea

**DOI:** 10.1186/s12884-024-06531-x

**Published:** 2024-05-14

**Authors:** Kamalini Lokuge, Freda Wemin, Grace Joshy, Glen DL Mola

**Affiliations:** 1 National Centre for Epidemiology and Population Health, The Australian National University, 62 Mills Road, Canberra, Acton, ACT 2601 Australia; 2 Goroka Provincial Hospital, 441, Eastern Highlands Province, PO Box 392, Goroka, Papua New Guinea; 3 School of Medicine and Health Sciences, University of Papua New Guinea, Papua New Guinea, NCD, Box 5623, Port Moresby, Boroko, Papua New Guinea

**Keywords:** Basic emergency obstetric and newborn care, Papua new guinea, Low- and middle-income countries (LMIC), Maternal and newborn health, Primary health care, Rural health centres

## Abstract

**Background:**

60% of women in Papua New Guinea (PNG) give birth unsupervised and outside of a health facility, contributing to high national maternal and perinatal mortality rates. We evaluated a practical, hospital-based on-the-job training program implemented by local health authorities in PNG between 2013 and 2019 aimed at addressing this challenge by upskilling community health workers (CHWs) to provide quality maternal and newborn care in rural health facilities.

**Methods:**

Two provinces, the Eastern Highlands and Simbu Provinces, were included in the study. In the Eastern Highlands Province, a baseline and end point skills assessment and post-training interviews 12 months after completion of the 2018 training were used to evaluate impacts on CHW knowledge, skills, and self-reported satisfaction with training. Quality and timeliness of referrals was assessed through data from the Eastern Highlands Province referral hospital registers. In Simbu Province, impacts of training on facility births, stillbirths and referrals were evaluated pre- and post-training retrospectively using routine health facility reporting data from 2012 to 2019, and negative binomial regression analysis adjusted for potential confounders and correlation of outcomes within facilities.

**Results:**

The average knowledge score increased significantly, from 69.8% (95% CI:66.3-73.2%) at baseline, to 87.8% (95% CI:82.9-92.6%) following training for the 8 CHWs participating in Eastern Highlands Province training. CHWs reported increased confidence in their skills and ability to use referral networks. There were significant increases in referrals to the Eastern Highlands provincial hospital arriving in the second stage of labour but no significant difference in the 5 min Apgar score for children, pre and post training. Data on 11,345 births in participating facilities in Simbu Province showed that the number of births in participating rural health facilities more than doubled compared to prior to training, with the impact increasing over time after training (0–12 months after training: IRR 1.59, 95% CI: 1.04–2.44, p-value 0.033, > 12 months after training: IRR 2.46, 95% CI:1.37–4.41, p-value 0.003). There was no significant change in stillbirth or referral rates.

**Conclusions:**

Our findings showed positive impacts of the upskilling program on CHW knowledge and practice of participants, facility births rates, and appropriateness of referrals, demonstrating its promise as a feasible intervention to improve uptake of maternal and newborn care services in rural and remote, low-resource settings within the resourcing available to local authorities. Larger-scale evaluations of a size adequately powered to ascertain impact of the intervention on stillbirth rates are warranted.

## Background

Papua New Guinea (PNG) has the highest maternal mortality rates in the Pacific region: estimates range from 145 to > 500 deaths per 100,000 live births [1, 2], but are likely to be at the higher end of this range [3]. PNG also has amongst the highest infant mortality rates, at 35.2 deaths per 1000 live births [4], and neonatal deaths (NNDs) account for nearly half of these infant deaths. An estimated two-thirds of these newborn (and some stillbirths) lives that are lost could be saved with basic but effective maternal and newborn care. The World Health Organization (WHO) recommends a package of services delivered through basic and comprehensive emergency obstetric care facilities for addressing the major causes of maternal and neonatal death and morbidity in and around the time of birth [5]. The main gap identified in provision of these services is at the basic health facility level, or in the delivery of basic emergency obstetric and neonatal care (BEmONC). BEmONC services are identified as a set of seven signal functions: administration of parenteral antibiotics; administration of uterotonic drugs; administration of parenteral anticonvulsants for preeclampsia and eclampsia; manual removal of the placenta; removal of retained products; assisted vaginal delivery; and basic neonatal resuscitation [5]. 

Many low and middle-income (LMIC) countries do not have the specialist workforce required to achieve adequate access to BEmONC services [6–8]. This is often coupled with delays or inability for women to reach health facilities when complications arise [9, 10]. In PNG, these challenges are compounded by geographic access barriers. 80% of PNG’s population reside in remote and rural regions with few health facilities or trained health professionals, contributing to low facility and supervised birth rates [3]. To effectively address these barriers to achieving adequate coverage of health workers with the expertise to provide appropriate emergency obstetric and neonatal services at basic health facility level, community-level approaches are urgently needed. Various approaches have been trialled across LMIC settings to address the lack of availability of adequate BEmONC and specialised staff (obstetricians, midwives, and medical practitioners with obstetric certification). These include supporting capacity-building within communities and primary health care facilities in rural or remote areas to allow for treatment of a wider range of obstetric complications, conditional cash transfers, incentives for supervised birth in the local rural health facility, and task-sharing with CHWs [11–13]. 

Numerous studies demonstrate the important role of CHWs in improving the quality and value of health care in a variety of settings, particularly those burdened by resource shortages [14, 15]. CHW-led interventions have been shown to be both effective and cost-effective for managing a range of health conditions among vulnerable populations [16, 17]. In PNG, CHWs (previously referred to as enrolled, maternal and child health or public health nurses) receive basic nursing training, and play a critical role in delivering health care to rural and remote populations [18, 19]. Upskilling of non-specialised CHWs has been a key component of the PNG government’s strategy to improve maternal and child health outcomes for over a decade. This has been reiterated in the National Health Plan 2021–2030, which identifies CHWs as essential to supporting sustainable community-based options for health promotion, awareness, and preventive activities [20]. 

This paper presents findings from an evaluation of a practical, hospital-based on-the-job training program implemented by local health authorities in PNG between 2013 and 2019 aimed at upskilling CHWs to provide quality maternal and newborn care in rural health facilities. The evaluation aimed to assess the impact of the CHW upskilling program on trainees’ knowledge, skills, and confidence in BEmONC service provision, and on the rural maternal and newborn care services in the Simbu province following CHW upskilling training.

## Methods

### Study population and facilities

The CHW upskilling program was implemented in several provinces, but this evaluation focussed on two bordering provinces: Simbu and Eastern Highlands. This program evaluation included both prospective and retrospective components. The prospective component was a training evaluation designed to assess skill development in a single cohort of trainees in the Eastern Highlands Province. The retrospective component was a pragmatic design to evaluate the impact of the training program on health service delivery outputs and impact from earlier rollouts of the program in Simbu Province, with indicators chosen based on routinely available health facility reporting data over this period.

The specific objectives of the evaluation in the Eastern Highlands Province were to: (1) measure changes in knowledge and skills of CHWs immediately after completion of training (compared to before training started); (2) assess CHWs self-reported knowledge, skills, and confidence in the provision of maternal and newborn care at the rural facility level and satisfaction with training at 12 months post completion of training; and (3) assess the quality and timeliness of referrals from facilities with staff who had received training. The specific objectives of the evaluation in Simbu Province were to assess whether the training resulted in (1) improved uptake and outcomes of maternal and neonatal care as indicated by birth and stillbirth rates; and (2) change in the number of referrals for maternal and neonatal patients requiring hospital services.

The original study aimed to compare quantitative health facility data from the year before training (2017) to the year after training (Sep 2018 - August 2019) in Eastern Highlands Province only. However, due to a polio outbreak in early 2019 in PNG, staff from all health centres, including those participating in the training program, were seconded for a 3-month period to support a catch-up mass immunisation campaign throughout the country. This limited the post-intervention data available prospectively in Eastern Highlands Province. The methodology was therefore revised to incorporate routinely collected health facility data for Simbu Province from 2012 to 2019, excluding data from the first quarter of 2019 to account for the impact of the immunisation campaign mentioned earlier.

### The CHW upskilling program

In 2008, a PNG Ministerial Task Force initiative established an in-service training program in Provincial Hospital Maternity Units (PHMUs) to improve maternal and newborn care. The initiative aimed to strengthen the ability of CHWs to provide quality maternal and newborn care (including BEmONC skills) in rural health centres, with a goal to improve the quality and coverage of BEmONC services in rural and remote health centres and ensure timely referrals for maternal and neonatal patients requiring hospital services. The CHW upskilling training program consisted of six months of primarily on-the-job training in obstetric and neonatal care at a provincial referral hospital supervised by the senior obstetrician alongside trained designated preceptor midwives staffing the labour wards and other clinical areas, along with classroom teaching for usually not more than one hour per day. The training content was based on a curriculum and training program developed by Professor Glen Mola (SMHS-UPNG) and Dr Lahui Geita (former Senior Maternal Care Advisor at the National Department of Health PNG) in conjunction with a number of senior midwives and CHW school trainers. Content covered key aspects of maternal and newborn care and included the WHO tenets of BEmONC (such as management of shock and post-partum haemorrhage, vacuum extraction, shoulder dystocia, bag and mask resuscitation of the newborn, manual removal of the placenta for retained placenta and use of MgSO4 for severe pre-eclamptic toxaemia and eclampsia prior and parenteral antibiotics for the treatment of sepsis prior to referral to hospital).

The program was developed and first implemented in Simbu Province and Western Highlands Provinces with six cohorts of trainees from 22 different facilities, 20 of which were rural health centres. Each cohort included approximately 8–10 CHWs. All cohorts completed training between 2013 and 2018, with one or two cohorts graduating per year. Two or three staff in each of the rural health centres are involved in maternal and newborn care, one of whom was selected for upskilling. Participating CHWs were required to have had at least five years of experience working in the maternal and newborn service in a rural health facility that was caring for at least 100 births per year, and be committed to returning to the facility to work after completion of training. The final selection of participating facilities and CHWs was carried out by the Family Health Services coordinator of the Provincial Health Authority. In 2015 the curriculum was approved by the National Health Curricula approval committee of the PNG National Department of Health.

With feedback received from the training in Simbu Province, the program was refined and then implemented in Eastern Highlands Province for the first time in 2018. The 2018 training in Eastern Highlands included eight CHWs from six rural and remote health centres alongside two CHWs from Goroka Hospital, the main referral hospital in the Eastern Highlands Province. The participating Eastern Highlands health centres were chosen as they were facilities where more than 100 women per annum came to seek supervised birth care and where there were recently improved facilities to provide supervised births.

This program has now been implemented in 14 provinces across PNG since its commencement in 2012. The cost of the 6-month program is approximately AUD 40,000 for 8–10 trainees, plus the salary contribution of the provincial health authority they are employed by during the 6 months in which they are on training leave (a further AUD 4000 per trainee). The Simbu, Eastern and Western Highlands Provincial Health Authorities (PHAs) have received funding support from a variety of NGOs and United Nations (UN) agencies to support the cost of training for six cohorts of trainees, and all PHAs in participating provinces funded trainees’ salaries for the 6-month training period. The additional costs of the program (aside from salary) for a further 13 cohorts were covered by various local charities and organisations.

### Objectives, indicators, and data sources

The indicators used to evaluate the specific program objectives are described in Table 1 below.

**Table 1:** Specific objectives and related indicators

Specific objectives	Evaluation indicators
Improvement in a sub-set of indicators related to the access, uptake and outcomes of basic emergency obstetric and neonatal care	
Service access: Increase in CHW skills in maternal & newborn care (including BEmONC) through a training program	Participating CHWs demonstrate improvements in pre and post training BEmONC skills assessment (Eastern Highlands Province) Participating CHWs self-report improved confidence and ability to apply new BEmONC skills at 12-month follow up interview. (Eastern Highlands Province)
Service uptake: Increased numbers of women presenting to facilities for supervised birth	Number of births in participating health facilities during study period. (Simbu Province)
Service outcomes	Number and proportion of stillbirths occurring in health centres, out of all births in participating health facilities during study period. (Simbu Province)
**Improved referral for maternal and neonatal patients requiring hospital services in rural areas**	
Improvements in timeliness and appropriateness of emergency obstetric and neonatal referral	Increase in proportion of referrals in first stage of labour from participating health facilities to the provincial hospital (Eastern Highlands Province) Increase in proportion of referrals for complex deliveries (e.g., Failure to progress in labour, Ante Partum Haemorrhage etc.). Improvement in 5-minute Apgars. (Eastern Highlands Province)
Strengthened referral pathways between health centre and referral hospital obstetric staff	Qualitative improvements in referral pathways between health centre and referral hospital reported by CHWs and obstetricians in the receiving referral hospital at the 12-month follow up interview. (Eastern Highlands Province)
The number of referrals	Number of referrals from participating health facilities during study period. (Simbu Province)

### Evaluation of CHW skill development in Eastern Highlands Province

All CHWs participating in the 2018 Eastern Highlands Province training participated in the evaluation. Participation involved completing a written skills assessment at baseline (prior to commencing training) and at completion of training to assess changes in skill level. This skills assessment was based on the JHPIEGO Maternal and Neonatal Health Program’s Guidelines for Assessment of Skilled Providers After Training in Maternal and Newborn Healthcare. This tool is based on WHO maternal and newborn care guidelines and has been utilised in a range of countries to assess skills development [21]. The assessment included 72 questions covering knowledge related to the management of antenatal care and family planning (10 questions each); normal labour, childbirth, and immediate newborn care (30 questions); postpartum care (mother and child) (10 questions); and management of complications of delivery (12 questions). Results were a simple tally of correct versus overall questions (as a percentage). Practical skills were assessed on the job by the midwife preceptor throughout the training period and clinical competence was signed off in a logbook at the conclusion of training.

The CHWs and the senior obstetrician at Goroka Hospital were also invited to participate in semi-structured interviews a year after the training was completed (October 2019) to assess perceptions about CHW knowledge, skills, and confidence in the provision of BEmONC services, satisfaction with the training program and how it could be further strengthened, and any challenges experienced in implementing the training.

### Evaluation of births, stillbirths, and referrals in Simbu Province

Data on facility births, stillbirths and referrals was included from a total of 20 rural health centres and sub-health centres in Simbu Province which had one trainee attend upskilling during the period from 2013 to 2018, with pre- and post-evaluations conducted using data from 2012 to 2019 to ensure pre and post intervention data was available from all facilities. Data from the first quarter of 2019 was excluded as during this period a national polio mass immunisation campaign was implemented which took most health centre staff away from their place of work for some (if not all) of this period, resulting in very limited activities within facilities.

Quarterly aggregate data on births, stillbirths, and referrals from health centres for the pre- and post-training periods between 2012 and 2019 were analysed from routine reporting data obtained through the Provincial Health Information System. Facility births were quantified as count data measured quarterly. Stillbirths and referrals were calculated as a percentage of total births.

### Evaluation of quality and timeliness of referrals in Eastern Highlands Province

The senior obstetrician and staff at Goroka Hospital extracted data from the hospital and from labour ward registers and referral forms for 2017–2019 (that is, pre- and post-training) from facilities that had staff who had participated in the 2018 training. These data were analysed to assess the quality and timeliness of referrals, including stage of labour on arrival, reason for referral and type of delivery. During in-depth interviews, CHW’s and staff at Goroka Hospital were asked to comment on the relationship between health centres and the referral hospital and the strength of the referral pathway prior to and after CHW upskilling.

### Analysis

The changes in CHW knowledge pre- and post-training (based on results of the written skills-assessments) were assessed using descriptive and basic comparative analyses. Interview data exploring CHW’s self-perceived knowledge, skills and confidence and their experiences post-training, and theirs and referral hospital staff experiences and views on referrals were analysed using thematic analysis.

For the quantitative analysis of Simbu Province facilities, each quarter of data contributed by a health centre was classified according to whether it was before, during or after return of staff from training. The post-training period was further divided into the first 12 months following training, and later than 12 months following training to assess any change in the impact of training over time. Given training was conducted at different periods for each facility, the dates of these periods varied by facility.

The changes in pre- and post-training facility level outcomes including number of births, and referral and stillbirth rates were assessed using negative binomial regression. We adjusted for seasonal variation as access to health facilities can be affected by weather in rural and remote areas. This variable was defined as “wet” (2nd and 3rd quarter of each year) and “dry” (1st and 4th quarter of each year), which very closely align with these seasons in PNG. We also adjusted for trends over time across the whole of the study period by using a variable that accounts for passage of time by quarter (the unit of our data) to account for factors such as change in population size. We used a random effects model that accounted for correlation of study outcomes within facilities. All quantitative analysis was conducted using STATA/MP 17.0 (StataCorp LLC).

### Ethics review and approval

Ethical approval for this evaluation was granted by the PNG Medical Research Advisory Committee and the Human Research Ethics Committee of the Australian National University (Protocol no. 2018/425). Written informed consent was obtained from participating CHWs.

## Results

A total of eight CHWs from six rural and remote health centres were trained over a six-month period in the Eastern Highlands Provincial Hospital in Goroka in 2018. In Simbu Province, there were a total of 540 quarters of observation included from the 20 health facilities in the study (i.e., Q1 2012 to Q3 2019, excluding Q1 2019). 280 of these quarters were in the pre-training period, 34 during training and 226 following training. There were 31 quarters where no births occurred, 21/280 (8%) of which were in the pre-intervention period. Throughout the post-training follow up period, only 9/226 (4%) of quarters across all facilities reported no births.

### CHW knowledge, confidence, skills, and experience, Eastern Highlands Province

Following the 2018 Eastern Highlands training, there was a significant improvement in CHW knowledge. At baseline (prior to commencing training) the average knowledge score was 69.8% (95% CI:66.3-73.2%). Following training, the average knowledge score achieved was 87.8% (95% CI:82.9-92.6%). Interviews at 12 months after completion of the training were completed with six of the eight CHWs from five participating health facilities, and with the senior obstetrician and staff at the Eastern Highlands provincial hospital.

#### Self-reported skills and confidence

All CHWs reported improved skills in a wider range of procedures and more confidence in patient care. They also reported more confidence in engaging with referral hospital health staff and professionals with regard to referral of patients and performing procedures in their rural health facility. CHWs are normally rostered on after-hours to manage deliveries and previously felt unskilled in this role. For example, CHWs would typically stay with women in labour throughout the night if needed until she gave birth, whereas other health centre staff (such as resident nurses and midwives) would usually go home at 5pm, asking the CHWs to “call if there is a problem.” They now felt more confident in this role.

CHWs also reported stronger referral networks because of improved personal connections with senior staff in the Provincial Hospital that were involved in their training. The strengthened relationships meant that CHWs were able to seek early advice from senior staff about cases who were developing complications thereby improve the timeliness and appropriateness of their referrals. This was also reported by the senior obstetrician and staff in the referral hospital, who stated that upskilled staff discussed possible referrals whereas those health centres and staff who had not participated in training often referred without discussion of the case, resulting in delayed and unnecessary referrals.

Despite these positive impacts, CHWs also indicated several health systems factors that could have attenuated the full impact of the upskilling, including poor or lack of water supply, electricity and equipment, frequent stock-outs of key medications in their health centres, and lack of transport to refer patients in a timely manner.

### Simbu facility births and birth outcomes

#### Facility births

In Simbu Province, 11,345 births in total occurred across the 20 rural health centres that participated in the upskilling over the study period. 4,147 of these births were in periods prior to upskilling of staff in those facilities, and 6,526 occurred after upskilling. (672 births occurred during the time health staff were receiving upskilling training). The number of births in study facilities more than doubled in the period following return of the staff member after completion of their training, when compared to the period prior to them departing for training, based on adjusted analysis accounting for change over time, seasonality of births and correlation within facilities. This increase was found to be statistically significant (see Table 2).

**Table 2:** Adjusted incidence rate ratio estimates for association between facility births and pre-or post-training period, Simbu Province, 2012–2019

Births	Incidence rate ratio	95% CI	*p*-value
Period relative to training			
• Pre-training (Reference)	1.0		
• During 6-month training period	1.312	0.933–1.847	0.119
• 0–12 months after training and return to HC	1.591	1.038–2.441	0.033
• > 12 months after training and return to HC	2.459	1.370–4.413	0.003
Season	1.0		
• Wet (Reference)	1.0		
• Dry	0.999	0.938–1.063	0.969
Time (by quarters, from Jan 2012 – to Sep 2019)	0.967	0.944–0.990	0.005

*Adjusted for time since commencement of study and season (wet/dry), and accounting for correlation of outcomes within facilities

#### Simbu province stillbirths

In Simbu Province, the rate of stillbirths in the participating health centres and sub-health centres did not vary significantly before and after upskilling in adjusted analysis (Table 3).

**Table 3:** Adjusted incidence rate ratio estimates for association between stillbirth rates and pre-or post-training period*, Simbu Province 2012–2019

Stillbirths	Incidence rate ratio	95% CI	*p*-value
Births	0.972	0.936–1.010	0.153
Period relative to training			
• Pre-training (Reference)	1.0		
• During 6-month training period	1.757	0.504–6.119	0.376
• 0–12 months after training and return to HC	1.352	0.430–4.247	0.606
• > 12 months after training and return to HC	1.412	0.290–6.880	0.669
Season			
• Wet (Reference)	1.0		
• Dry	0.824	0.420–1.615	0.573
Time (by quarters, from Jan 2012 – to Sep 2019)	0.960	0.918–1.004	0.078

*Adjusted for total births in period, time since commencement of study and season, and accounting for correlation of outcomes within facilities

The stillbirth incidence per 1000 births calculated from the adjusted analysis above for the pre- and post-training periods are given in Table 4 below.

**Table 4:** Stillbirth incidence rates (and confidence intervals) per 1000 births based on the incidence rate ratios from the adjusted analysis presented in Table 3

Stillbirths	Rate/1000 births	95% CI	*P*-value
Pre-training	16.07	8.14-24.00	0.000
During 6-month training period	28.23	0-57.69	0.060
0–12 months after training and return to HC	21.72	0-42.69	0.042
> 12 months after training and return to HC	22.70	0-54.97	0.168

The between and within facility variance in stillbirth rates based on our data is 0.173 and 2.342 respectively. The intra-cluster correlation coefficient calculated from these variances is 0.069. The estimated design effect for stillbirth rates due to clustering within these 20 facilities is 1.999.

#### Simbu province referrals

In Simbu Province, the rate of referrals in the participating health centres and sub-health centres did not vary significantly before and after upskilling in adjusted analysis (Table 5).

**Table 5:** Adjusted incidence rate ratio estimates for association between referral rates and pre-or post-training period*, Simbu Province 2012–2019

Referrals	Incidence rate ratio	95% CI	*p*-value
Births	0.954	0.908–1.001	0.0.060
Period relative to training			
• Pre-training (Reference)	1.0		
• During 6-month training period	0.753	0.407–1.396	0.368
• 0–12 months after training and return to HC	0.735	0.352–1.535	0.412
• > 12 months after training and return to HC	0.521	0.166–1.635	0.264
Season			
• Wet (Reference)	1.0		
• Dry	0.966	0.619–1.507	0.879
Time (by quarters, from Jan 2012 – to Sep 2019)	0.998	0.966–1.030	0.885

*Adjusted for total births in period, time since commencement of study and season), and accounting for correlation of outcomes within facilities

Based on the above adjusted analysis, the rate of referrals was 21 per 1,000 births prior to upskilling, and 15.5 and 11.0 per 1,000 births respectively at 0–12 months and > 12 months following upskilling.

#### Timeliness and appropriateness of referrals

Table 6 presents the characteristics of referrals from 2017 to August 2019 to the Eastern Highlands provincial hospital from health centres participating in the 2018 CHW upskilling. There were significant increases in births arriving in the second stage of labour following training, but no significant difference in the 5 min Apgar score for children born pre and post training.

**Table 6:** Characteristics of the 832 referrals to Goroka Hospital from participating health centres in Eastern Highlands Province from 2017 to August 2019 (excluding Jan-Mar 2019 data) *

	2017	2018	2019 Apr-Aug	*P*-value^$^
**Total**	**402**	**192**	**127**	
Stage of labor on arrival**				
• Not in labor	43 (11%)	19 (10%)	13 (11%)	
• First	293 (73%	162(84%)	81(67%)	
• Second	61 (15%)	10 (5%)	19 (16%)	0.002
Type of birth				
• Spontaneous vaginal birth	315 (79%)	139 (72%)	81 (64%)	
• Birth requiring intervention assistance for maternal or newborn benefit	87 (27%)	53 (28%)	45(37%)	0.006
• missing	1 (0%)	0	1 (0%)	
Apgar’s at 5 min				
• 10	319 (81%)	146 (78%)	95 (79%)	
• < 10	77 (19%)	42 (22%)	25 (21%)	0.714
• missing	6 (1%)	4 (2%)	7 (6%)	
Who referred				
• Unknown/Missing	348 (87%)	132 (69%)	57 (45%)	
Reason for referral				
• Unknown/Missing	196 (49%)	77 (40%)	16 (12%)	

* % calculated by excluding missing values; **Calculated excluding those not pregnant on referral, no missing values; ^***$***^Fishers exact p-value calculated excluding missing values, for those variables with < 10% missing values

Data on the specific reason for referral were collected retrospectively from hospital records, and the large number of missing values in the 2017–2018 period for this variable limited the inferences that can be drawn. If only 2019 data are considered, the pattern of referrals during and after training appeared similar, with obstructed labour, high risk pregnancy, birth requiring intervention assistance for maternal or newborn benefit, and preeclampsia toxemia (PET) being the most frequent reasons for referral (data not shown).

## Discussion

CHWs in PNG undertake a two-year pre-service training, but this includes minimal curriculum and training in maternal and newborn care unlike other staff such as midwives and nurses. However, when they are deployed to a rural health facility they are often assigned to the maternal and newborn care section of the facility. The training program was designed to address this skills gap. The community health worker upskilling program in the Eastern Highlands Province was well-received by participating staff, who reported that it increased their confidence and skills in managing labour and birth within their primary health care facilities. These results are consistent with prior evaluations of similar programs which demonstrate improvements in participants’ knowledge and skills that persist longer-term [22–27]. Evaluation findings further indicate improved links between these facilities and provincial level hospitals, which CHWs and staff used both to improve the timeliness of referrals and to obtain advice on complicated births. Facility-level indicators (increased births at facilities) from participating facilities in Simbu Province also showed improvements that may be attributable to the CHW upskilling program. Increases in births were higher in the period greater than 12 months following training than in the period within 12 months of training, despite no significant upward trend in births overall over time. Reasons for increase in supervised births over time following training may include that the systems barriers (e.g., lack of equipment, electricity, running water) the CHWs identified in their 12 month follow up interviews were subsequently addressed, and that the upskilled CHWs passed on their skills to other staff in the facility. It may also be that the facilities were increasingly recommended within communities due to positive feedback from women who had used the facility and were happy with the care received. Alternatively, other policy or program changes outside of the upskilling program may have contributed the uptake of care in facilities. However, we are unaware of any specific such programs during the that were implemented selectively in the post-intervention period of study facilities, noting also that the dates of the post-intervention period varied by facility. Any external programs would have been implemented at provincial level over different time frames to the upskilling and are therefore unlikely have contributed to the specific increase in births we found pre and post intervention.

Emergency referrals are an important mechanism for improving outcomes for time-critical conditions that may occur during pregnancy, birth, and the postnatal period. Delayed or inappropriate referrals have been identified as important contributing factors to adverse maternal obstetric and neonatal outcomes [28, 29]. Both the health centre and referral hospital staff reported qualitative improvement in the referral pathway between these two levels of care and in the timeliness and appropriateness of referrals.

Stillbirths levels are important in informing policy and program design, however there is limited evidence of program impact on stillbirth rates in LMICs [30]. There is some evidence that comprehensive interventions focusing on improving emergency obstetric and neonatal care services may be effective in decreasing stillbirth rates in low-income contexts [31]. However, our study found no statistically significant difference between the rate of stillbirths in the participating health centres and sub-health centres before and after upskilling in adjusted analysis. A similar stillbirth rate to that identified in this evaluation was reported in a recent study of stillbirths in two provinces in PNG between 2017 and 2019 (23 per 1000 births) [32], reinforcing the need to address high stillbirth rates in PNG and include stillbirths data in future research. However, one confounding factor with regards to the numbers of stillbirth recorded at participating rural health facilities might be that before the upskilling training, CHWs routinely referred women with fetal death in utero (FDIU) to hospital, whereas care of the FDIU cases was part of the upskilling training, and CHWs were capacitated to care for women with FDIU in their own rural health facility.

The implementation of the CHW upskilling program reveals the potential of this training to address the lack of access to high-quality maternal obstetric services in much of PNG. Continuing education programs for midwives, nurses, and other health professionals such as the upskilling program may also improve care quality and possibly retention of health staff [33–38], which is another major challenge for the PNG health system.

Implementing this program alongside other interventions that incentivise facility births in rural health facilities has the potential to further enhance its impact. A program providing incentives such as ‘baby bundles’ and livelihood generating tools for fathers has been implemented by members of the study team in selected health facilities in the study provinces since the completion of this study, and appears to show promise [39]. Co-implementation of incentivising and upskilling interventions could improve program outcomes, however, further research is needed.

Our results indicate that system-level barriers such as facility infrastructure and insufficient supplies have a notable impact on trainees’ capacity to implement the skills acquired from the training program. Thus, addressing these health-system factors would be critical to optimising its effectiveness. The widespread rollout of this program across 14 provinces of PNG, despite the significant cost-constraints under which the PNG health budget operates, is a strong indicator that it is feasible within the resources available to the Provincial Health Authorities that managed its implementation in each of the participating provinces.

Implementation of any program should be done alongside robust evaluation processes to contribute evidence of effective, generalisable interventions for maternal and neonatal health [40], and this study adds further to this evidence base. Given the critical shortage of health staff in rural areas, and the supportive and close relationship between the formally recognised roles of CHWs and midwives/nurses in rural areas, upskilling of CHWs is a core strategy for increasing availability of high-quality birthing services in rural areas within the framework of the PNG health system. In PNG, CHWs are all certified to supervise birth and the national supervised birth proportion is calculated to include those births in facilities supervised by a CHW. In general, health workers in PNG are legally permitted to perform procedures that they have been trained to do. The CHW Upskilling training is approved and certified training program by the National Dept of Health Curriculum and Training certification committee. CHWs and Nursing officers work side by side in rural health facilities providing maternal and newborn care; however, the nursing officer often takes on a more supervisory role and the CHWs actually perform the maternal and newborn care as there are very few trained Registered Nurse midwives practicing in rural health (level 3 facilities) centres in PNG. The CHW Upskilling training in the provincial hospitals is largely carried out by midwives preceptors, who have been involved in the formulation of the trainings and generally are very supportive of it. Midwives have reported on the positive aspects of their experiences in upskilling groups of CHWs to the annual midwifery scientific meetings.

### Limitations

We acknowledge a number of limitations that should be accounted for when reviewing these results. These include that the health facility-level indicators were chosen based on data available retrospectively through routine reporting, and do not represent the full comprehensive set of indicators for assessing quality, uptake, and coverage of BEmONC services. However, these indicators have been selected for routine reporting because they are important measures of facility performance in the areas targeted by the training program, namely access to, quality and outcomes of emergency obstetric care. A further limitation in making inferences from this study is that the 12-month follow up assessment was based on qualitative self-report by participants, unlike the pre and post skills assessment. This raises the possibility of bias in the longer-term assessment of program impact. Our quantitative results demonstrated that birth rates increased in the period > 12 months compared with the period < 12 months after training, suggesting no attenuation of training impact over time in regard to this indicator. As previously discussed, it is unlikely this effect would be due to other external programs. However, to increase confidence in this finding, including for other indicators outside of births, we recommend that future evaluations include a follow-up skills assessment by supervisors on site, or preferably prior to short-term refresher courses conducted at the provincial hospital at regular intervals.

We did not have exact staff numbers in each facility to adjust for proportion trained relative to staffing. However, the numbers are similar across participating health facilities (2–3 involved in provision of maternal and newborn care) and remained constant over time when comparing the pre- and post-training periods. Given our analysis compared change over these periods within each facility and included a random effect parameter accounting for other factors that may have varied between facilities, any differences in staffing number or proportion of trained staff between facilities would have been adjusted for.

Data from Simbu Province on which stillbirth calculations were based included a large number of facility births (over 11,000). Despite this, the relatively small number of stillbirths meant that our study was insufficiently powered to detect smaller changes in these rates. To determine in a future study whether the mean difference seen in this study was statistically significant, based on our findings we would need to include over 21,000 births. Further, upskilled CHWs no longer referred all fetal deaths in utero (FDIU) to the provincial hospital. Prior to upskilling, these fetal deaths in utero were referred, and therefore recorded as a stillbirth in the hospital data rather than at the facility. Thus, it is likely that the stillbirth rate prior to training is an underestimation of the actual stillbirths in the rural health facilities but we lacked data to assess this. Accounting for stillbirth referrals in future studies is important, including determining the number of facility referrals for fetal deaths-in-utero.

Finally, qualitative findings indicate that there were various health system factors that are likely to have attenuated the full impact of the upskilling program. However, these factors could not be adjusted for in the quantitative analysis. Further qualitative investigation is warranted to improve understanding of these health system factors and their impacts on service births in the PNG context.

Our study did not include a comprehensive set of BEmONC indicators, as defined by the WHO [5]. Future prospective research, based on a broader set of routinely collected facility data, will allow a more comprehensive understanding of the impact of our intervention on quality and coverage, and of services for pregnant women more broadly. Additionally, understanding the perspectives of women who use these services is an essential aspect of evaluation, one which was not possible in this study, but which is critical to include in future studies.

## Conclusions

Our findings showed positive impacts of the upskilling program on CHW knowledge, skills and confidence of participants, facility birth rates, and appropriateness of referrals, demonstrating its promise as a feasible intervention to improve uptake of maternal and newborn care services in rural and remote, low-resource settings within the resourcing available to local authorities. Larger-scale evaluations of a size adequately powered to ascertain impact on stillbirth rates are warranted. It is essential that future roll outs are implemented alongside interventions to ensure that systems requirements (adequate structures, electricity, running water, equipment etc.) are in place to effectively implement the skills acquired during training.

## Data Availability

The data that support the findings of this study are available from the National Department of Health (Papua New Guinea), but restrictions apply to the availability of these data, which were obtained with permission for the current study, and so are not publicly available. Data are however available from the lead author (Kamalini.Lokuge@anu.edu.au) upon reasonable request and with the permission of the National Department of Health (Papua New Guinea).

## Abbreviations

BEmONC: Basic emergency obstetric and neonatal care
CHW: Community health worker
FDIU: Fetal deaths in utero
NNDs: Neonatal deaths
PET: Preeclampsia toxemia
PHAs: Provincial Health Authorities
